# Questionnaires based on natural language processing elicit immersive ruminative thinking in ruminators: Evidence from behavioral responses and EEG data

**DOI:** 10.3389/fnins.2023.1118650

**Published:** 2023-03-06

**Authors:** Yulong Li, Chenxi Li, Tian Zhang, Lin Wu, Xinxin Lin, Yijun Li, Lingling Wang, Huilin Yang, Diyan Lu, Danmin Miao, Peng Fang

**Affiliations:** ^1^Department of Military Medical Psychology, Air Force Medical University, Xi'an, China; ^2^Key Laboratory of Military Medical Psychology and Stress Support of PLA, Xi'an, China; ^3^Shaanxi Provincial Key Laboratory of Bioelectromagnetic Detection and Intelligent Perception, Xi'an, China; ^4^School of Biomedical Engineering, Air Force Medical University, Xi'an, China

**Keywords:** natural language analysis, immersive questionnaire, EEG coherence, psychological selection, rumination

## Abstract

Rumination is closely related to mental disorders and can thus be used as a marker of their presence or a predictor of their development. The presence of masking and fabrication in psychological selection can lead to inaccurate detection of psychological disorders. Human language is considered crucial in eliciting specific conscious activities, and the use of natural language processing (NLP) in the development of questionnaires for psychological tests has the potential to elicit immersive ruminative thinking, leading to changes in neural activity. Electroencephalography (EEG) is commonly used to detect and record neural activity in the human brain and is sensitive to changes in brain activity. In this study, we used NLP to develop a questionnaire to induce ruminative thinking and then recorded the EEG signals in response to the questionnaire. The behavioral results revealed that ruminators exhibited higher arousal rates and longer reaction times, specifically in response to the ruminative items of the questionnaire. The EEG results showed no significant difference between the ruminators and the control group during the resting state; however, a significant alteration in the coherence of the entire brain of the ruminators existed while they were answering the ruminative items. No differences were found in the control participants while answering the two items. These behavioral and EEG results indicate that the questionnaire elicited immersive ruminative thinking, specifically in the ruminators. Therefore, the questionnaire designed using NLP is capable of eliciting ruminative thinking in ruminators, offering a promising approach for the early detection of mental disorders in psychological selection.

## Introduction

Rumination is defined as continuous attention to negative stimuli, including the causes and consequences of negative events and the resulting negative emotions, which may lead to depression, anxiety, and other mental disorders. This phenomenon is considered a non-adaptive way of regulating emotions (Nolen-Hoeksema, 1991; Nolen-Hoeksema and Morrow, 1991; Nolen-Hoeksema et al., 1995). In the Self-Regulatory Executive Function (S-REF) model of emotional disorders, Wells and Matthews identified a persistent thinking mode that included worry and rumination and further defined it as an ineffective coping strategy. Rumination involves persistently focusing on negative thinking and feelings rather than problem solving, which is counterproductive by way of amplifying and prolonging the experience of suffering (Wells and Matthews, 1996). The relationship between rumination and depression has been widely studied (Castanheira et al., 2019; Li et al., 2020; Van Doorn et al., 2021). According to response styles theory, rumination can prolong and intensify the pain of negative or stressful events, increase despair, and aggravate depressive symptoms (Nolen-Hoeksema et al., 2008). Rumination is also associated with alcohol abuse, anxiety symptoms, generalized anxiety disorder, social anxiety disorder, obsessive-compulsive disorder, PTSD, schizophrenia, borderline personality disorder, and bulimia nervosa, among others, and other mental disorders (Watkins and Roberts, 2020).

Although there are numerous scales available for assessing rumination, the most widely used remains the Ruminative Response Scale (RRS), developed by Nolen-Hoeksema in 1991 (Nolen-Hoeksema and Morrow, 1991) and compiled by Nolen Hoeksema in 1991. While RRS has been widely employed in ruminative research, the limitations of this scale are also significant, especially regarding the presence of psychological selection (Wang et al., 2020; Miao et al., 2021). Camouflage, falsification, social approval, and other issues (Dunning et al., 2005; Schwarz, 2012; Wang et al., 2020, 2021) have greatly affected the acceptance and validity of rumination-related psychological testing. Therefore, further advancements are necessary in inducing ruminative conscious activity accurately, measuring the associated brain activity objectively, and exploring the neural biomarkers of rumination to identify ruminators and predict future rumination for precise psychological selection.

Previous studies have found that rumination arises as a result of negative situational memories (Sutherland and Bryant, 2007). Hence, the resurfacing of specific situational memories is considered one of the most effective and rapid means to induce rumination. In episodic memory-related studies, Wilson Mendenhall proposed “scenario immersion” and defined it as precise language that could facilitate immersive psychological imagination and enable subjects to place themselves into various imagined situations and memories (Wilson-Mendenhall et al., 2019). After experiencing different emotions, subjects form an episodic memory in their long-term memory, thus affecting the emotions they will experience in similar situations in the future. For example, when someone perceives that a car is approaching them quickly in a given scenario, their episodic memory from a previous similar scenario is activated in response. This implicitly, rapidly, and synergistically generates fearful cognitive, interoceptive, and behavioral processes in relation to the current situation (Lebois et al., 2020). Research has shown that rumination can be activated quickly when encountering situations that are similar to a past fearful event or when encountering only a small component of the original event (Wilson-Mendenhall et al., 2015). In 2022, Priyamvada Rajasethupathy confirmed the core idea of the “scenario immersion” theory with the finding that a holistic episodic memory composed of multiple sensory experiences can indeed be evoked by a single sensory cue (Yadav et al., 2022). Therefore, eliciting a specific episodic memory can be the most effective and efficient means to initiate ruminative thinking (Sutherland and Bryant, 2007).

Natural language (i.e., the language used in daily life) is an important means of human communication and an essential feature that distinguishes human beings from other animals (Assale et al., 2019). A specific questionnaire based on natural language can be used to comprehensively induce the recall of the complete episodic memory (Wilson-Mendenhall et al., 2019; Ancin-Murguzur and Hausner, 2021). At present, natural language processing (NLP) technology has accomplished a series of complex functions, such as machine translation, automatic summarization, emotion analysis, and text classification (Castanheira et al., 2019). Compiling questionnaires based on natural language corpuses and NLP of rumination are the two methods that are considered effective in inducing and analyzing rumination accurately (Ferrario et al., 2020). The theory of “scenario immersion” is a useful tool for inducing rumination; therefore, we drew upon this theory and further incorporated NLP to develop a questionnaire that could elicit ruminative thinking both efficiently and effectively.

Compared to cognitive neural technologies, such as functional magnetic resonance imaging (fMRI), magnetoencephalography (MEG), and functional near-infrared spectroscopy (fNIRS), wireless EEG technology is cheap, easy to transport, convenient for recording brain activity during experiments, sensitive to the alternation of brain activity, flexible across various experimental paradigms, and provides high temporal resolution signals, thereby rendering it suitable for language evaluation and the detection of brain activity during psychological selection (Deshpande et al., 2017).

EEG coherence, first proposed by Robinson (Robinson, 2003), is an index of brain connectivity that is calculated by the covariance of the power spectral density at two electrodes. Coherence shows the synchronicity of neural activity and reflects brain dynamics (Markovska-Simoska et al., 2018). Neuroimaging studies have provided a great deal of data on the dysfunction and dysregulation that occurs in the brains of clinically and nonclinically depressed ruminators, including hypofunctional and hyperfunctional connectivity (Ferdek et al., 2016; Li et al., 2018; Benschop et al., 2020). Such studies were designed to examine network characteristics while ruminators were at rest or engaged in a task, thereby exposing the synchronicity of neural activity either between the brain areas (Ferdek et al., 2016) or between the brain networks (Zhang et al., 2020). Both EEG and fMRI studies have shown that altered functional connectivity in the brain networks of subjects with depression was positively related to ruminative thoughts (Benschop et al., 2020). These results indicated that the altered synchronicity within the brain might be an indicator of altered brain activity. EEG coherence may be a suitable indicator to depict the change of the brain activities in subjects with mental disorders.

In this study, we developed a situational ruminative questionnaire using the natural language characteristics of ruminators to demonstrate the ability of the questionnaire to elicit immersive ruminative thinking in ruminators, as determined by their behavioral responses and characteristics of EEG signals.

## Methods

### The ruminative response scale

The Ruminative Response Scale (RRS) is a questionnaire composed of 22 items that is used to measure the ruminative tendency of an individual. Each item uses a 4-point Likert scale (1 = rarely, 2 = sometimes, 3 = often, 4 = almost always). The Chinese version used in this study was translated by Han Xiu and has been verified to have good reliability and validity among Chinese senior high school and college students (Han and Yang, 2009). The higher the total score, the higher the rumination level, and the highest score is 88. The demographic information of the participants is shown in Table 1.

**Table 1:** Demographic information of 4,591 participants (*n* = 4,591).

	**Frequency/mean**	**Percentage/standard deviation**
**Gender (** ***n*** **, %)**		
Women	65	1.42
Men	4,526	98.58
**Age (mean** **±SD)**	21.29	2.59
**Home location (** ***n*** **, %)**		
Urban	1,191	25.94
Village	3,400	74.06
**Ethnicity (** ***n*** **, %)**		
Han	4,109	89.50
Others	482	10.50

### Natural language processing and questionnaire development

We selected 607 subjects with a high tendency for rumination (577 men and 30 women) for semi-structured interviews that were conducted on an individual basis. The interviews were recorded and saved, and an intelligent conference system (version 5.0) was used to transcript the interview information for each interviewee. The demographic information of the participants is shown in Table 2. The obtained transcripts were analyzed, and the elicited material was compiled using the following six steps. (1) Proofreading and denoising: The text was thoroughly analyzed to check for errors and delete any extraneous characters, spaces, and so on. (2) Chinese participle: Chinese word segmentation was performed using Chinese LIWC software. (3) Stop word filtering: More uniform word segmentation text was created using the HIT edition of the “Stop Word Dictionary” to filter stop words. (4) Feature word extraction and coding: The TF-IDF approach was used to determine the frequency of feature words in the text (Reviewer-Lee, 2000; Jurafsky, 2009). The feature words were specifically selected by using the TF-IDF algorithm to calculate the frequency of all the words in the interview transcript of the extreme ruminators to obtain the high-frequency feature words first and then by referring to the 66 “scenario materials” words in Mendenhall's article (Wilson-Mendenhall et al., 2019). Only the content words (to construct the scenario) and the depictive words (to express the emotional experience) of the feature words were retained, and function words with no real meaning were removed. The bag-of-words model used a real-valued vector to label each transcribed word and encoded it using feature words (Ferrario et al., 2020). (5) Based on the semantic characteristics of the feature words, the LDA (latent Dirichlet allocation) topic model was used to extract text topics (Ghosh and Guha, 2013; Min et al., 2019, 2020), which were then used to group words under the topics. The detailed analysis pipeline of the LDA model and the document generation process are shown in Supplementary Figure 1 (Blei et al., 2003; Hao et al., 2017). Quantitative analysis was then used to identify the overarching topic of the interview material, while artificial naming was used to identify the situational topics. An example of extracting text topics is shown in Supplementary Table 1. (6) Creation of situational inducing materials: We recreated real-life scenarios that the interviewees had described, merged them with the six syntaxes of Mendenhall's “scenario immersion” theory, and then generated materials that elicited rumination. The neutral items were derived from paragraphs that were cut from the third edition of the Encyclopedia of China, whereby the number of words was kept similar to that of the rumination items and the chosen content was relatively boring and meaningless.

**Table 2:** Demographic information of individuals who participated in the semi-structured interviews (*n* = 607).

	**Frequency/mean**	**Percentage/standard deviation**
**Gender (** ***n*** **, %)**		
Women	30	4.94
Men	577	95.06
**Age (mean** **±SD)**	21.80	3.03
**Home location (** ***n*** **, %)**		
Urban	196	32.29
Village	411	67.71
**Ethnicity (** ***n*** **, %)**		
Han	525	86.49
Others	82	13.51

### Questionnaire evaluation

The ruminative questionnaire was revised by three linguistics professors. The revisions made included connotation logic, grammar application, and character norms, among others. To verify the validity of the questionnaire regarding its ability to induce rumination, it was evaluated again by high- and low-degree ruminators. We then recruited another 1,685 subjects (all males) to complete the RRS. Of these, 78 participants were randomly selected for the final evaluation of the developed questionnaire. These participants comprised 40 high-degree ruminators (ruminators, mean age = 23.30 years, SD = 3.40 years; RRS score: 53.65 ± 8.89) and 38 low-degree ruminators (controls, mean age = 20.84 years, SD = 1.55 years; RRS score: 22.13 ± 0.34). We based the degree of rumination on the cutoff values specified in the Rosenbaum et al. (2018b) article: high-degree ruminators were defined as having a mean RRS score higher than 2.36 (PR > 65), while low-degree ruminators were defined as having an RRS score lower than 1.9 (PR < 27) (Rosenbaum et al., 2018b). Neutral items were also evaluated in comparison with the rumination items. The evaluation included seven dimensions: (1) repetition; (2) persistence; (3) associativity; (4) vividness; (5) uncontrolled nature; (6) assumption; (7) representativeness. The first six dimensions represent ruminative characteristics summarized from both our literature review and from interviews conducted with a large number of highly ruminative individuals. The last dimension, representativeness, reflects the extent to which an entry matched the subject's recall. These seven dimensions combine to represent the level of “scenario immersion” experienced by subjects while they were filling out the questionnaire. The demographic information of the 1,685 and 78 participants is shown in Tables 3, 4, respectively.

**Table 3:** Demographic information of the 1,685 participants (*n* = 1,685).

	**Frequency/mean**	**Percentage/standard deviation**
**Gender (** ***n*** **, %)**		
Women	0	0
Men	1,685	100
**Age (mean** **±SD)**	21.19	1.60
**Home location (** ***n*** **, %)**		
Urban	308	18.28
Village	1,377	81.72
**Ethnicity (** ***n*** **, %)**		
Han	1,489	88.37
Others	196	11.63

**Table 4:** Demographic information of the 78 participants who participated in the evaluation of the questionnaire (*n* = 78).

	**Frequency/mean**	**Percentage/standard deviation**
**Gender (** ***n*** **, %)**		
Women	0	0
Men	78	100
**Age (mean** **±SD)**	22.10	2.92
**Home location (** ***n*** **, %)**		
Urban	33	42.31
Village	45	57.69
**Ethnicity (** ***n*** **, %)**		
Han	71	91.03
Others	7	8.97

### EEG experimental procedures

#### EEG experiment participants

We recruited subjects from the 1,685 freshmen enrolled in the 2022 cohort of Shaanxi Police College to participate in the EEG experiment. Finally, 56 voluntary participants (mean age = 22.48 years, SD = 7.73 years) selected from the high-degree ruminators (PR > 65, RRS score: 63.79 ± 6.38) were recruited as the rumination group, while 29 voluntary participants (mean age = 20.59 years, SD = 1.64 years) (PR < 27, RRS score: 22.14 ± 0.44) were recruited as the control group. The exclusion criteria were as follows: (1) patients with a history of psychiatry; (2) patients who had been hospitalized in the psychiatric department; (3) patients with current or past use of antipsychotic drugs; (4) patients with a history of neurological disorders; (5) patients who were left-handed. The study was approved by the Ethics Committee of the Air Force Medical University and was conducted in accordance with the approved guidelines (Ethics Approval Number KY20193304-1). All participants provided their informed consent prior to undergoing the formal experiment and received a small amount of compensation.

#### Resting-state EEG data acquisition

Resting-state EEG data were collected for all subjects before the test. First, the subjects were instructed to sit down in front of a screen in a comfortable position, with their eyes distanced ~70 cm from the stimulating screen. When the subjects were ready, the experimenter instructed them to close their eyes for more than 1 min and then to open them for more than 1 min.

#### Task EEG data acquisition under material stimulation

After recording resting-state EEG data, the subjects were instructed to complete the exam using the 53-item questionnaire (containing 37 rumination items and 16 neutral items). Items were displayed on the screen randomly, with a fixed 500-ms plus sign separating each stimulus from the next. All items presented the same following question: “Does the above description induce you to have repeated/continuous recall?”. The subjects selected “yes” or “no” as their response to this question using the mouse or keyboard. There were no time constraint for how long subjects were allowed to respond to each question. The item inquiry was completed as soon as the subject clicked the mouse to answer the questions.

#### EEG recording and data preprocessing

A 32-channel semi-dry electrode cap was used to record EEG data using a wireless multi-channel EEG acquisition device (ZhenTec NT1, ZhenTec Intelligence, China) (Yuan et al., 2021; Han et al., 2022). The sampling rate was 500 Hz. Data were referenced to CPz with a ground at FPz, and electrode placement followed the International 10-10 system. Impedance levels were set at < 20 kΩ. The common mode rejection ratio was 120 dB, the input impedance was 1 G, and the input noise was < 0.4 uVrms for all EEG channels.

For each subject, more than 2 min of resting-state EEG signals were recorded under two conditions: with eyes closed and with eyes open, with each condition lasting more than 1 min. Additionally, while participants completed the test items, EEG signals were acquired. All of the EEGs were pre-processed prior to the commencement of further research using the FildTrip (Version 20221122) (Oostenveld et al., 2011) toolbox implemented in MATLAB 2018b. We first checked the quality of the data and processed the band channel using the interpolation method. Both the resting state and task signals were notched by 50 Hz to remove power-line interference. Then, the EEGs were band filtered with a 1–100 Hz zero-phase band filter. Subsequently, the signals were divided into two 1-min epochs for the eyes-closed and eyes-open conditions for resting-state EEGs. We segmented the task EEGs into each item-related signal epoch based on the time markers of eye movements where signals were acquired simultaneously (53 items and 53 task signals for each participant in each channel). We manually checked and removed any data with large interference. The data were considered invalid if the faulty segments totaled more than five. The task EEG analysis was performed on 75 subjects—the average EEG length of the ruminator.

The remaining EEG data were then subjected to independent component analysis (ICA) to determine brain signals. Based on the spatial distribution and spectral power, FildTrip was used to find the independent components (ICs), including motor activity, eyeblinks, and ECG, which were then eliminated prior to further analysis.

### EEG power analysis

After preprocessing, both the eyes-closed and eyes-open resting state signals were filtered into theta, delta, alpha, and beta bands. Then, we employed the “pwelch” function in MATLAB to calculate the spectral power during the resting state for all 30 channels in each of the four frequency bands: delta (0.5–4 Hz), theta (4–8 Hz), alpha (8–13 Hz), and beta (13–30 Hz).

### Coherence analysis

Referring to the analysis method of a previous study by Bakker, we also adopted the coherence method to reflect the alternation in brain activity (den Bakker et al., 2018). The task EEG signals were then filtered into theta and gamma frequency bands (4–80 Hz). The bands were theta (4–8 Hz), alpha (8–13 Hz), beta (13–30 Hz), gamma1 (30–60 Hz), and gamma2 (60–80 Hz). Subsequently, we employed the “mscohere” function in MATLAB to calculate magnitude-squared coherence, which reflects how well signal *x* corresponds to signal *y* at each frequency band. The “mscohere” function estimates the magnitude-squared coherence function using Welch's overlapped averaged periodogram method (we used 512 points per window with 90% overlap). The coherence value of signals *x* and *y* *C*_*xy*_(*f*) was calculated as a function of the following: the spectral densities of signal *x*, which was denoted *as P*_*xx*_(*f*); the spectral densities of *y*, which was denoted as *P*_*yy*_(*f*); and the cross spectral density of x and y, which was denoted as *P*_*xy*_(*f*):


$$\begin{array}{l} C_{xy}\left(f\right)=\frac{|P_{xy}\left(f\right){|}^{2}}{P_{xx}\left(f\right)P_{yy}\left(f\right)} \end{array}$$

Then, the coherence values were averaged across all pairs of electrodes over the brain for further analysis. We also calculated the average coherence in each band. To minimize the effects of volume conduction, we set the coherence of neighboring electrodes to zero before calculating the average coherence in each band (Peters et al., 2013; den Bakker et al., 2018).

### Correlation between the coherence of EEGs and the arousal rate

The arousal rate of each participant induced by the rumination items was calculated as the proportion of “yes” responses given to the total number of rumination items. Finally, we adopted the Pearson correlation method to investigate the relationship between brain EEG coherence in different frequency bands and the arousal rate induced by the rumination items.

### Statistical analysis

Both the behavior results and EEG characteristics were analyzed statistically using SPSS 26 (IBM). To examine the behavioral difference between the two groups across the two item types in each of the seven dimensions, a two-sample *t-*test was utilized. To determine the power differential of the resting-state EEGs in each frequency band, we utilized a two-way repeated ANOVA using the group and channel as factors. Mauchly's test was applied to test for sphericity, while the Greenhouse–Geisser correction was used to correct the sphericity. The coherence in the five bands was then compared, both in-group and item-wise. The statistical method was also referred to in the previous study (den Bakker et al., 2018). For group-wise comparison, we first employed a two-way repeated ANOVA with the item type and band as factors to compare the averaged coherence across the brain. The difference between the rumination items and the neutral items was determined using the two-sample *t*-test, and Bonferroni's correction was used for multiple comparisons (*p* < 0.01). We then adopted a two-way ANOVA using item type and frequency as factors to assess their differences in each frequency band (theta, alpha, beta, gamma1, and gamma2). For item-wise comparison, we set the group and band as factors and performed the same statistical procedure.

## Results

### Demographic information of the participants

Table 1 shows the demographic information of the 4,591 subjects who used the RRS. In this study, the total average score of the RRS was 31.57 ± 8.33 (*n* = 4,591), and Cronbach's α coefficient for this scale was 0.925 (*n* = 4,591).

Table 2 shows the demographic information of the 607 high-degree ruminators (577 men and 30 women) who participated in the semi-structured interviews.

Table 3 shows the demographic information of the 1,685 subjects. In this part, the total average score of the RRS was 28.64 ± 6.97 (*n* = 1,685), and Cronbach's α coefficient for this scale was 0.925 (*n* = 1,685).

Table 4 shows the demographic information of the 78 chosen subjects from the 1,685 pool of subjects who completed the questionnaire.

Table 5 shows the demographic information of the 75 participants who were recruited for the following EEG experiment.

**Table 5:** Demographic information of the 75 participants who participated in the EEG study (*n* = 75).

	**Frequency/mean**	**Percentage/standard deviation**
**Gender (** ***n*** **, %)**		
Women	0	0
Men	75	100
**Age (mean** **±SD)**	21.75	6.18
**Home location (** ***n*** **, %)**		
Urban	23	30.67
Village	52	69.33
**Ethnicity (** ***n*** **, %)**		
Han	66	88.00
Others	9	12.00

### Natural language feature adaptation paradigm

**Figure 1:**
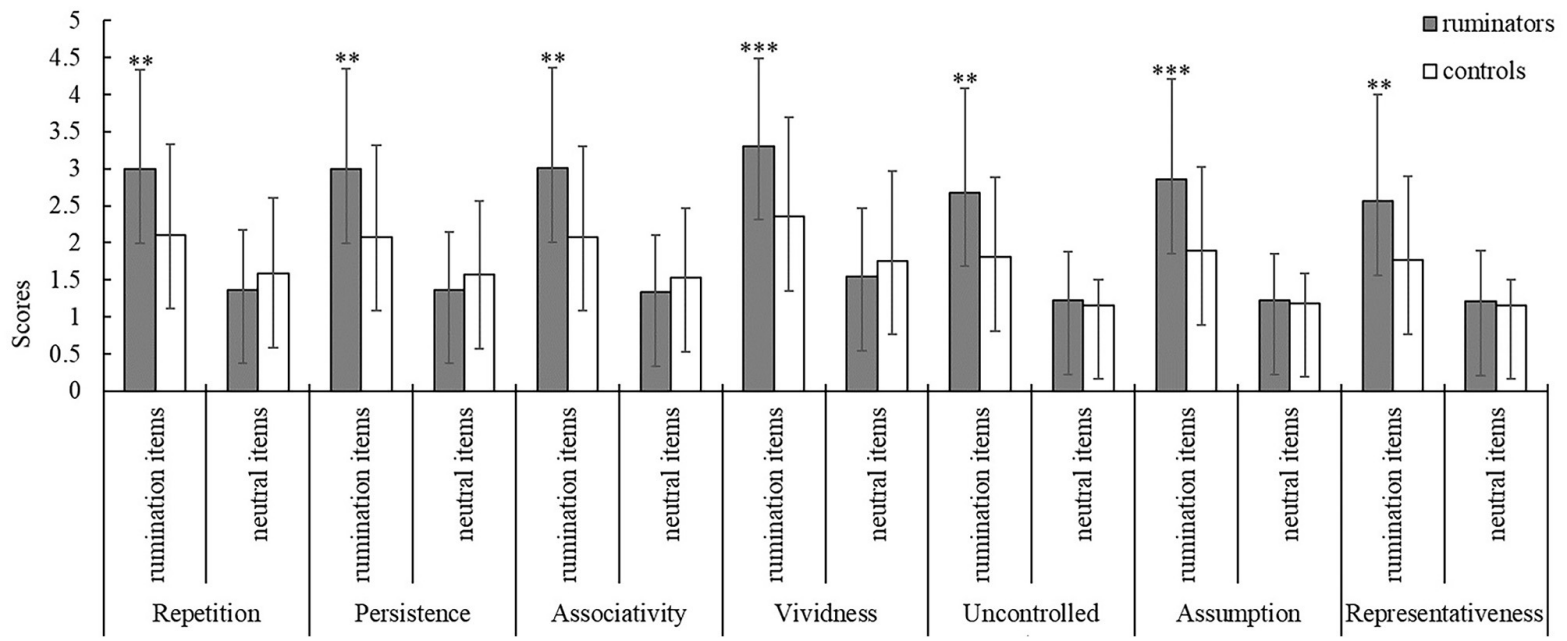
Comparison of ruminator and control subject scores on the rumination and neutral items under the seven dimensions. **p < 0.05, ***p < 0.001, error line represents the standard deviation.

Using NLP techniques, we identified 17 ruminative scenario themes and constructed 37 ruminative arousal-inducing items (including 28 social dilemmas and 9 personal injuries). The validation results for the rumination questionnaire showed that the main effects of the item types were significant in all dimensions (*p* < 0.001). Further simple effect analysis showed that the ruminators' scores on rumination items were higher than those of the control group (*p* < 0.05), while the rumination group scores of the neutral items were no different from those of the control group (Figure 1). This result confirmed the effectiveness and reliability of the rumination items.

### The reaction time was longer in ruminators for rumination items

The ANOVA of the two groups and item type revealed that the main effect of the two groups was not significant [*F*_(1, 73)_= 0.792, *p* = 0.377, η*p*^2^ = 0.11], while the main effect of the item type was significant [*F*_(1, 73)_ = 107.968, *p* < 0.001, η*p*^2^ = 0.597], reflecting that the reaction time of rumination items was longer than that of the neutral items. Additionally, a significant interaction between item type and person category was found [*F*_(1, 73)_ = 31.901, *p* < 0.001, η*p*^2^ = 0.304]. Therefore, further simple effects analysis was conducted, and the results are as follows (Table 6).

**Table 6:** Response times ($\bar{x}\;$ ± SD) of the ruminators and the controls (NC) on both item types.

**Type of item**	**Ruminators (*n* = 46)**	**Controls (*n* = 29)**	***t***	***P***
Rumination	15.731 ± 4.624	12.823 ± 3.143	−3.240	< 0.01
Neutral	9.663 ± 4.045	11.028 ± 3.524	1.494	0.139

The results of the simple effects analysis showed that the response time of the ruminators while engaging with rumination items was longer than that of the control group (*df* = 73, *p* < 0.01), while the response time of the ruminators while engaging with neutral items did not differ from that of the control group. This result further verified the effectiveness of the ruminative questionnaire in inducing the ruminators to immerse themselves in and resonate with the different scenarios established by the questionnaires.

### The arousal rate was significantly higher in ruminators for rumination items

In the questionnaire response task, whether for the rumination items or the neutral items, we set the same following question: “Does the above description induce you to have repeated/continuous recall?”. This question reflects the core definition of the concept of rumination. Therefore, we interpreted the choice of “yes” to mean that the participants were aroused by the scenario. Conversely, participants who chose “no” were not considered to be aroused.

Arousal rate: the arousal rates elicited by the two item types were calculated as the proportion of the given response “yes” to the total for each kind of item (i.e., the number of “yes” responses given in rumination items/the total number of rumination items).

The main effect of the two groups was found to be significant, *F*_(1, 73)_ = 32.554, *p* < 0.001, η*p*^2^ = 0.308, and the arousal rate of the ruminators was higher than that of the control group. The main effect of the item type was also significant, *F*_(1, 73)_ = 82.300, *p* < 0.001, η*p*^2^ = 0.530, and the arousal rate for the rumination items was higher than that for the neutral items. The main effect of the item type and group was found to be significant, *F*_(1, 73)_ = 82.300, *p* < 0.001, η*p*^2^ = 0.530, and was higher than that of the neutral items. A significant interaction of the item type with the two groups was found [*F*_(1, 73)_ = 46.382, *p* < 0.001, η*p*^2^ = 0.389]. Therefore, further simple effects analysis was conducted, and the results are as follows (Table 7).

**Table 7:** Arousal rates ($\bar{x}\;\pm$ SD) of the ruminators and controls on both item types.

**Type of item**	**Ruminators (*n* = 46)**	**Controls (*n* = 29)**	***t***	***P***
Rumination	0.616 ± 0.324	0.158 ± 0.187	−7.746	< 0.001
Neutral	0.079 ± 0.146	0.082 ± 0.123	0.095	0.925

The results of the simple effects analysis showed that, for the rumination items, the arousal of the ruminators was significantly higher than that of the control group (*df* = 73, *p* < 0.001), while for the neutral items, the arousal of the ruminators did not differ from that of the control group. This finding indicates that the rumination items constructed in this study targeted the recall of the rumination group and thus triggered continuous and repeated recall.

### The brain power of normal subjects and high-degree rumination subjects were highly similar

**Figure 2:**
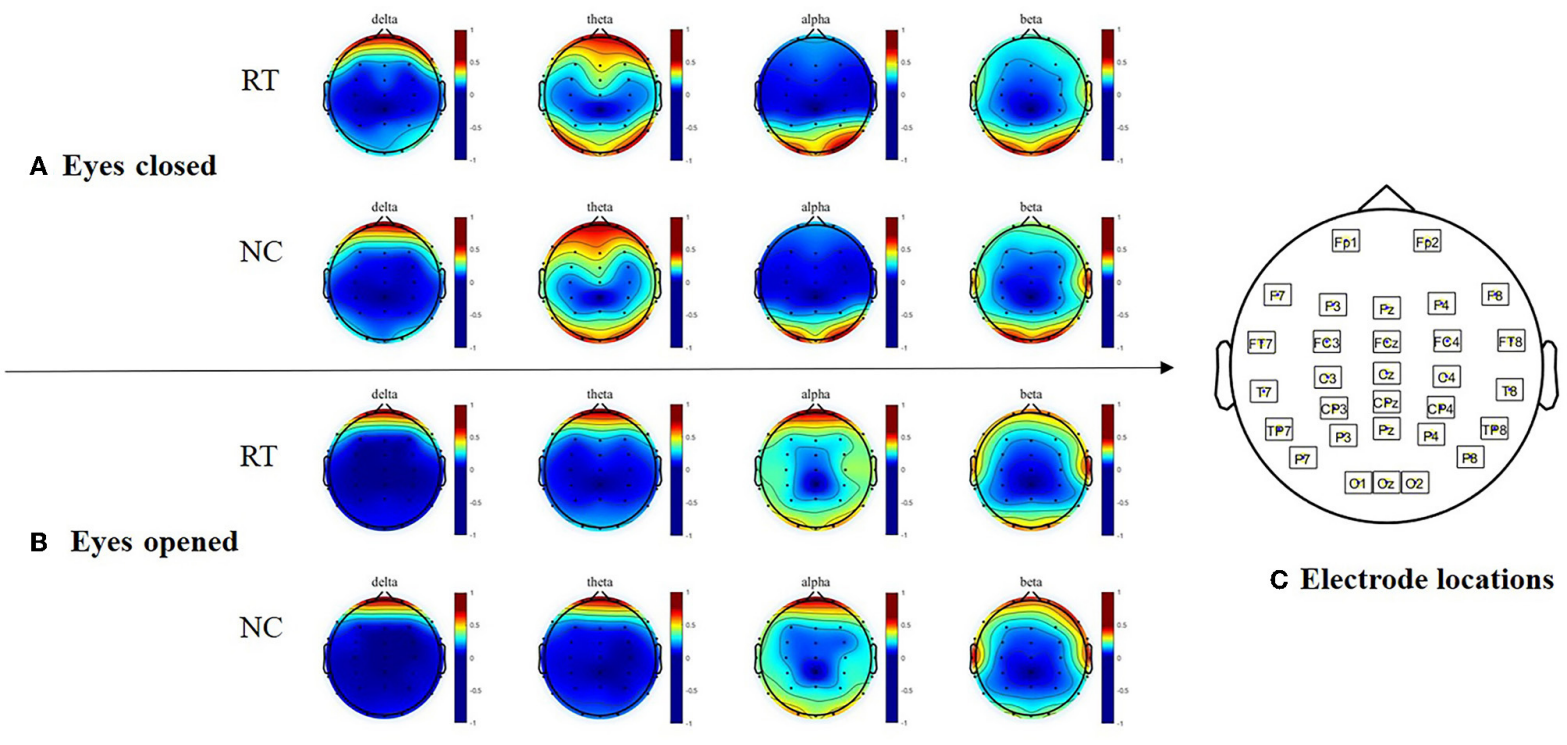
Topographical maps of absolute power in resting state EEGs. **(A)** Topographical maps of absolute power with eyes closed. **(B)** Topographical maps of absolute power with eyes opened. **(C)** Electrode locations of the EEG cap. RT, Rumination group; NC, control group.

The topography of the two groups in terms of the absolute power of each frequency band is depicted in Figure 2. In both the eyes-closed and eyes-open conditions, the two-way ANOVA did not reveal any group differences.

### The coherence of the whole brain was decreased while engaging with the rumination items

Table 8 shows the average EEG length of each group in different kinds of items. No significant differences were found in the length of EEG data either in groups or items.

**Table 8:** Average EEG length (time points) of the two types of items in different groups ($\bar{x}\;\pm$ SD).

**Type of item**	**Ruminators (*n* = 46)**	**Controls (*n* = 29)**
Rumination	6,750 ± 1,993	7,124 ± 2,146
Neutral	6,695 ± 2,274	6,709 ± 1,963

**Figure 3:**
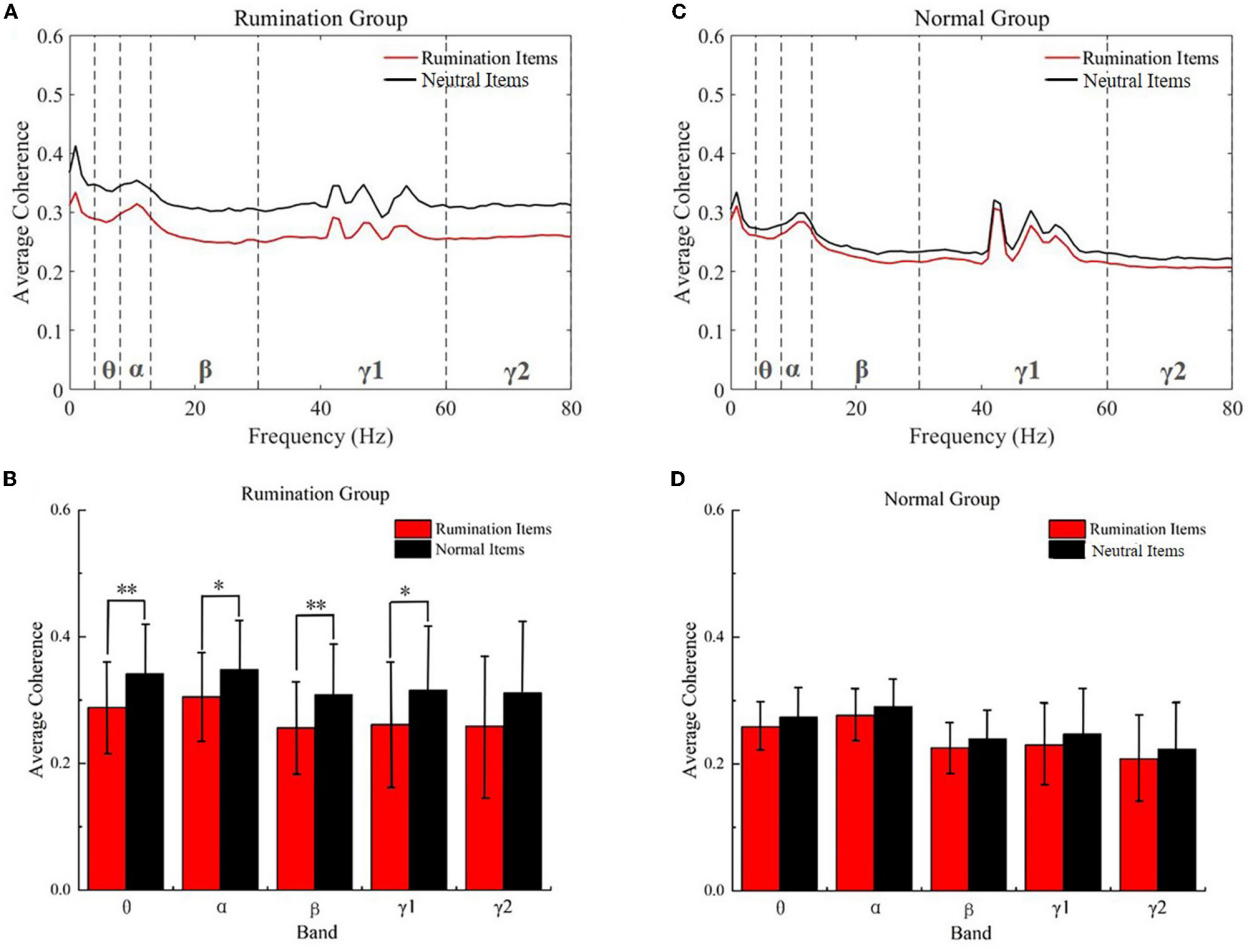
Within the group coherence analysis, the subjects completed the rumination items and neutral items. **(A)** Average coherence of all frequency bands in the rumination group. **(B)** Average coherence for the five frequency bands in the rumination group. **(C)** Average coherence of all frequency bands in the controls. **(D)** Average coherence in the five frequency bands in the controls. *p < 0.01, **p < 0.001. Red, rumination items; black, neutral items; Normal Groups means control groups.

As shown in Figure 3, the coherence of the whole brain was decreased in both groups while engaging with the rumination items compared to the neutral items in the test. In the rumination group, the two-way repeated ANOVA revealed a significant main effect on the item type [*F*_(1, 90)_ = 9.445, *p* = 0.003]. Additionally, a no item × frequency band interaction effect was found [Greenhouse–Geisser corrected, *F*_(1.452, 130.724)_ = 0.271, *p* = 0.691]. All the two-sample *t*-tests for *post hoc* analysis satisfied the test of homogeneity of variance (*p* > 0.05) and revealed that the coherence decreased significantly in the theta (two-tail, *t* = −3.373, *df* = 90, *p* = 0.001), alpha (*t* = −2.827, *df* = 90, *p* = 0.006), beta (*t* = −3.287, *df* = 90, *p* = 0.001), and gamma1 (*t* = −2.649, *df* = 90, *p* = 0.01) bands. In the subjects from the control group, there was no significant effect of the item type [*F*_(1, 56)_ = 1.724, *p* = 0.195] and no item type × frequency band interaction effect [Greenhouse–Geisser correction, *F*_(1.583, 88.654)_ = 0.015, *p* = 0.967]. The two-sample *t*-test showed no significant change in the coherence in the five bands while engaging with the two kinds of items in the normal subject group.

### The coherence of the whole brain increased in the rumination group

**Figure 4:**
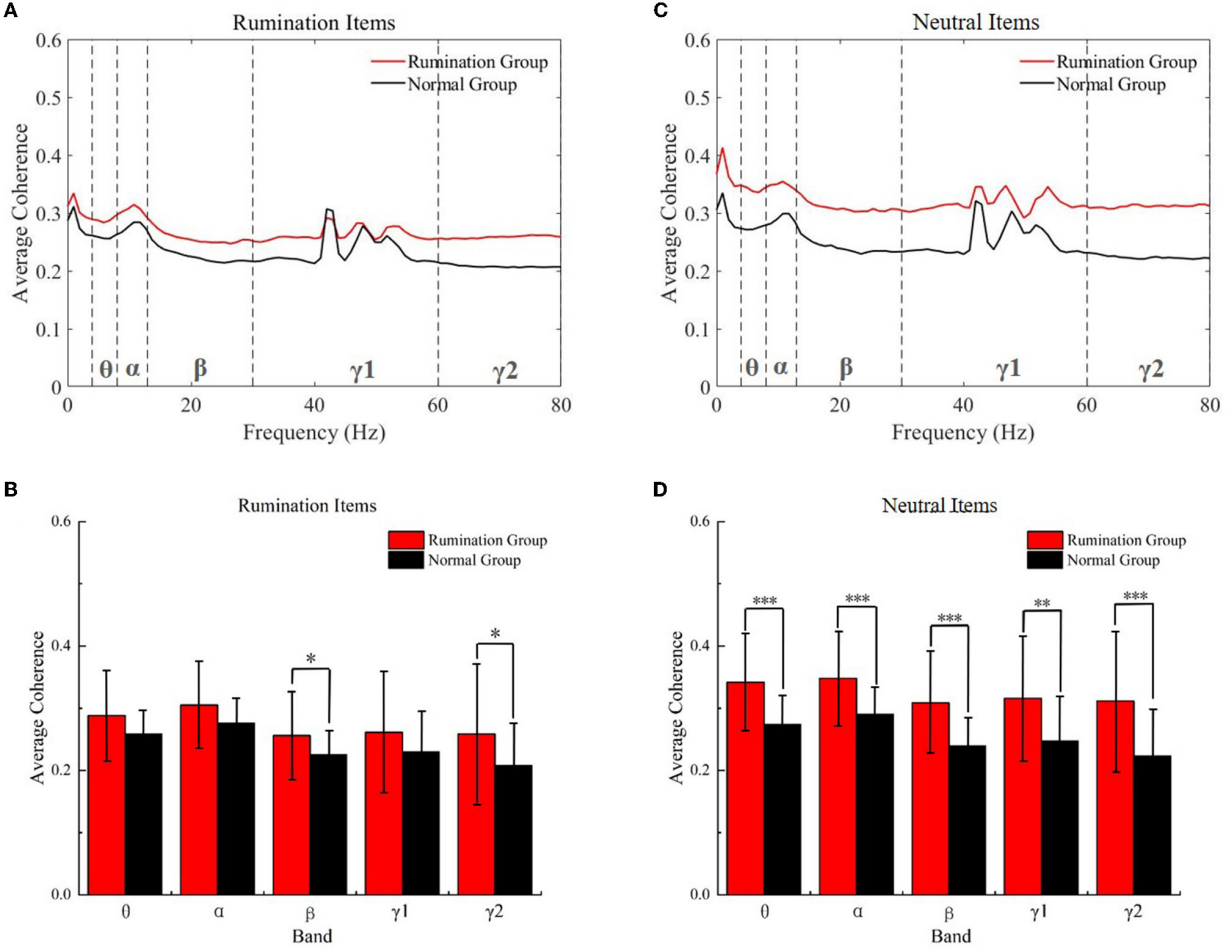
Within-item coherence analysis of the two groups while they engaged with the rumination items and neutral items. **(A)** Average coherence of all frequency bands in the rumination items. **(B)** Average coherence of the five frequency bands in the rumination items. **(C)** Average coherence of all frequency bands in the neutral items. **(D)** Average coherence of the five frequency bands in the neutral items. *p < 0.01, **p < 0.001, ***p < 0.001. Red, rumination items; black, neutral items; Normal Groups means control groups.

Next, we assessed the difference in coherence between the subjects from both groups while they were engaged with the two kinds of items. As shown in Figure 4, the coherence of all frequency bands increased [main effect of group *F*_(1, 73)_ = 4.916, *p* = 0.030, no group × frequency band interaction effect, *F*_(1.496, 109.172)_ = 0.834, *p* = 0.407, Greehouse-Geisser corrected], especially for the beta (two-tail, *t* = 2.301, *df* = 71.088 *p* = 0.017) and gamma2 (two-tail, *t* = 2.411, *df* = 72.953, *p* = 0.018) bands while the subjects were engaged with the rumination test. It is worth noting that the two groups showed a significant difference while interacting with the neutral items [main effect of group *F*_(1, 73)_ = 16.844, *p* < 0.000, no group × frequency band interaction effect, *F*_(1.538, 112.238)_ = 1.467, *p* = 0.235, Greehouse-Geisser corrected]. As Figure 4D shows, significantly increased coherence emerged in all frequency bands (two-tail: theta, *t* = 4.698, *df* = 72.949, *p* < 0.000; alpha, *t* = 4.160, *df* = 72.224, *p* < 0.000; beta, *t* = 4.684, *df* = 72.210, *p* < 0.000; gamma1, *t* = 3.446, *df* = 71.754, *p* = 0.001, and gamma2, *t* = 4.057, *df* = 72.662, *p* < 0.000) in ruminators engaging with the neutral items compared to the normal subjects. This phenomenon may have been due to the decreasing speed of the ruminative mood. This will be discussed later.

### The arousal rate was only significantly correlated with the coherence of the gamma2 frequency band

We conducted a Pearson correlation analysis to identify the relationship between the arousal rate induced by the rumination items and the EEG coherence of different frequency bands. We only found a significant correlation for the gamma2 band (*r* = 0.231, *p* = 0.046).

## Discussion

In this study, we first developed an immersive rumination-inducing questionnaire that could elicit situational recall in ruminators using NLP combined with scales, interviews, and “scenario immersion” theory. The results of behavioral indicators revealed that, for the rumination items of the questionnaire, the average reaction time was longer for the ruminators, and their arousal rate was significantly higher than that of the subjects in the control group. Then, we used EEG techniques to investigate whether the immersive ruminative questionnaire could induce certain neural activities, specifically in the ruminators. The resting-state EEG results showed that there was no difference in the power of the brain between the ruminators and the control group. However, the EEG coherence analysis showed that the brain activity of the ruminators was significantly higher than that of the control group for the rumination items. Combined with the behavior result and EEG evidence, the immersive ruminative questionnaire developed with natural language features and the scenario immersion theory was successful in eliciting more immersive ruminative thinking, specifically in the ruminators. In conclusion, the questionnaire based on NLP appears to be suitable as a novel paradigm for psychological selection in the early detection of mental disorders.

### The immersive ruminative questionnaire elicited immersive ruminative thinking, specifically in the ruminators

Previous studies have used various methods to induce rumination, including short statement prompts (Cooney et al., 2010; Berman et al., 2014; Milazzo et al., 2016), texts extracted from Wikipedia (Curci et al., 2015), characteristic words (Yoshimura et al., 2009; Moran et al., 2014; Apazoglou et al., 2019), music clips (Figueroa et al., 2017), self-reports (Rosenbaum et al., 2018a), emotive facial expressions (Aker et al., 2014), goal prompting tasks (Zhan et al., 2017; Mollaahmetoglu et al., 2021), videos (Bostanov et al., 2018), and other materials. However, it remains unclear whether these methods successfully induce subjects' rumination. Natural language is the first means by which humans express their thoughts and rapidly communicate accurately (Sun et al., 2013; Lee et al., 2014). Written materials pertaining to specific episodic memories represent one of the most effective forms of content to induce rumination (Haque et al., 2014; Wilson-Mendenhall et al., 2019). Rapidly developing NLP technology and “scenario immersion” theory provide us with technical support and a theoretical basis for constructing state-inducing questionnaires.

In this study, we used the natural language corpus of 607 ruminators and NLP to develop a mental state-eliciting questionnaire that could elicit situational memories according to “scenario immersion” theory to activate the unique ruminative state of rumination-prone individuals both effectively and accurately. The evaluation results showed that the scores of ruminators were significantly higher than those of the control group only on the rumination items in all dimensions. On the contrary, there was no significant difference between the neutral items. Therefore, the results indicate that the ruminators agreed with the scenario description constructed by the rumination items, which could not only trigger repeated, vivid, and continuous negative immersive memories but was also highly representative of ruminative elicitation. In other words, the questionnaire we developed could elicit immersive ruminative thinking in ruminators.

### The rumination items were capable of inducing brain activity in ruminators

The analysis of EEG power in the four frequency bands revealed no significant difference between the ruminators and the control group while they were at rest, indicating that there was no difference in brain activity between the ruminators and the control group in this state, which might be a contributing factor to the difficulty of diagnosing depression during its nonclinical state.

EEG coherence is a measure of synchronization of the two recorded EEG signals and has been widely used to indict the dysregulation of the human brain (den Bakker et al., 2018; Minami et al., 2022; Wang et al., 2022). We examined the task EEG data from both the item type aspect and group aspect to study if the different items could elicit different brain activities in the two groups. First, we discovered that EEG coherence decreased in both groups while subjects engaged with the rumination items. The ruminators presented a significantly greater decrease in the theta, alpha, beta, and gamma frequency bands. Although a slight decrease was also observed in the control group, no statistically significant differences were identified for any of the frequency bands. EEG coherence reflects the synchronization between brain cortical regions (Markovska-Simoska et al., 2018), whereby the observed significant decrease in the coherence of the whole brain indicates that the rumination items caused more dyssynchronization of the whole brain in the ruminators, thereby demonstrating that the questionnaire was capable of inducing ruminative thinking in these subjects. We also discovered that the EEG coherence of the ruminators dramatically increased both in the beta and gamma2 bands compared to the control group while engaging with the rumination items. In the ruminators, this aberrantly elevated coherence may indicate a decreased inhibitory ability of the brain, which might be caused by a depressed state of mind (Cheng et al., 2016), excessive self-focus, and the recall of unpleasant memories that the rumination items elicited (Berman et al., 2011; Zamoscik et al., 2014). The hyper-synchronization in the beta band combined with the longer reaction time suggests that the attention ability was damaged in the ruminators (Li et al., 2017). As the EEG gamma band has been shown to be related to emotions (Li et al., 2015), the increased coherence observed in the gamma2 band provides evidence that the rumination items induced an unpleasant mood in the ruminators. Finally, we found that the coherence of the whole brain in the five frequency bands increased significantly, which can be explained by the phenomenon where the influence of the rumination items did not subside immediately in the ruminators but instead was prolonged to influence the brain activity of these subjects while they were engaged with the neutral items. This also indicates a decline in their ability to control the brain after engaging with the rumination items.

The above findings showed that ruminators are more susceptible to negative scenarios, such as poor memories, bad moods, and inaccurate self-referential thinking. Over the past several years, studies have been conducted to identify the neurological bases of rumination in both clinical and nonclinical psychological diseases with the goal of developing potential biomarkers for the diagnosis and therapy of rumination-related mood disorders such as depression (Zhang et al., 2020), euthymic bipolar disease (Apazoglou et al., 2019), posttraumatic stress disorder (Philippi et al., 2020), and so on. Prior fMRI and EEG studies revealed that rumination is associated with altered brain functional connectivity, both increased (Benschop et al., 2020) and decreased (Tozzi et al., 2021). According to some studies, ruminators process self-related information excessively when exposed to external, which may be related to the overactive core subsystem in the default mode network (Lin et al., 2022), particularly the functional connectivity to the prefrontal cortex, which can be a useful neural marker to identify an individual at risk for depression (Benschop et al., 2020). Consistent with the findings of previous studies, we found that the EEG coherence of the whole brain increased significantly in the ruminators, suggesting that, through situational immersion, the questionnaire we developed can elicit excessive processing of self-related information in the high ruminators. This result indicates the validity of the questionnaire we developed. However, more research is required to determine the neurological mechanisms underlying the brain alteration that the questionnaire elicited, i.e., the prefrontal cortex activity and the relationship between different brain networks, which might be used as a neural marker of ruminative thinking.

## Limitations

There are some limitations to this study. First, all the participants were men due to the nature of the police academy. Although it is widely acknowledged that women tend to ruminate more than men do, one review reported that both men and women showed strong and significant statistical correlations between depressive symptoms and ruminative thoughts (rho > 0.50; *p* < 0.05), suggesting that the relationship between depressive symptoms and rumination does not necessarily explain the sex differences observed in depression (Shors et al., 2017). Hence, further study is required to explore the responses of women. Moreover, since all the subjects were enrolled in the Shaanxi Police College, more subjects will be needed from the general population in the following experiment. Second, the EEG evidence of ruminative thoughts from the perspective of the entire brain was the focus of this study, while specific brain regions or brain networks should be considered to identify the neuromechanism underlying rumination. Finally, neural biomarkers of rumination may be used to predict ruminators, which would be of great significance in psychological selection. In the future, we will explore the predictive power of the rumination items and EEG data.

## Data availability statement

The original contributions presented in the study are included in the article/Supplementary material, further inquiries can be directed to the corresponding authors.

## Ethics statement

The studies involving human participants were reviewed and approved by Ethics Committee of the Air Force Medical University. The patients/participants provided their written informed consent to participate in this study.

## Author contributions

YuL contributed to the development of the questionnaire and completed the whole experiment. CL analysis the EEG data and wrote the manuscript. TZ and LWu contributed to revision of the manuscript. XL, YiL, and LWa helped to analyze the questionnaire behavior data. DL and HY contribute big work to the experiment. DM and PF organized and managed the whole experiment and manuscript. All authors contributed to the article and approved the submitted version.
