# Supplementary material for: Questionnaires based on natural language processing elicit immersive ruminative thinking in ruminators: Evidence from behavioral responses and EEG data
Source: Front Neurosci. 2023 Mar 6;17:1118650. doi: 10.3389/fnins.2023.1118650 (PMC10025410; doi:10.3389/fnins.2023.1118650)
Supplement: Supplementary file 1 [file Data_Sheet_1.ZIP › Supplementary Tables.docx]

**Supplement Table 1. LDA topic model for extracting text topics**

| **Topic** | **Topic words** | **Summarizing topics** |
| --- | --- | --- |
| Topic1 | Disappointment, examination, physical test, exam results, failing grades, fall behind, inferiority complex, everyone, neglect, impatience, make friends, reaction, helplessness, denigration, face, obvious, indifference | Being ostracized or left out by classmates |
| Topic2 | Grandmother, father, cough, bad news, answering the phone, lorry, joining the police academy, mother, illness, crying, life, accident, lost, year round, mind, memories, paralysis | Serious illness or unexpected death of a family member |
| Topic3 | Monitor, evaluation award, unfair, anger, angry, news, physical performance, classmates, feel, class, belong to, deserve, relationship, dissatisfaction, intolerable, complaint | Suffering from injustice |

**Supplement Table 2. 17 Ruminative scenario topics**

| **Topic** | **Scenario topics** |
| --- | --- |
| Topic1 | Being ostracized or left out by classmates |
| Topic2 | Serious illness or unexpected death of a family member |
| Topic3 | Suffering from injustice |
| Topic4 | Public humiliation brings embarrassment |
| Topic5 | Failed and devastated romance |
| Topic6 | Insulted and verbally abused in public |
| Topic7 | Conflict or clash with superiors |
| Topic8 | Experiencing domestic violence |
| Topic9 | Accidental personal injury |
| Topic10 | Self-abuse or self-harm |
| Topic11 | Bullying at school |
| Topic12 | Experiencing the suicide of a friend or family member |
| Topic13 | Lack of close friends leads to inner loneliness |
| Topic14 | Excessive guilt or a heavy heart burden |
| Topic15 | Low self-esteem or lack of confidence |
| Topic16 | Inability to adapt to the current environment |
| Topic17 | Interpersonal difficulties |

**Supplement Table 3. Average number of time point of each item across subjects in ruminators.**

| **Item No.** | **Mean**$\boldsymbol{\pm}$**SD** | **Item No.** | **Mean**$\boldsymbol{\pm}$**SD** | **Item No.** | **Mean**$\boldsymbol{\pm}$**SD** |
| --- | --- | --- | --- | --- | --- |
| 1 | 8968$\pm$4104 | 21 | 11331$\pm$5178 | 41 | 6059$\pm$5290 |
| 2 | 6586$\pm$3114 | 22 | 9403$\pm$5037 | 42 | 8675$\pm$4094 |
| 3 | 9227$\pm$4496 | 23 | 7345$\pm$2020 | 43 | 6018$\pm$4274 |
| 4 | 7052$\pm$3283 | 24 | 6513$\pm$4404 | 44 | 6879$\pm$3542 |
| 5 | 5368$\pm$3428 | 25 | 5594$\pm$3415 | 45 | 6708$\pm$3325 |
| 6 | 7560$\pm$3717 | 26 | 7686$\pm$3406 | 46 | 5806$\pm$3693 |
| 7 | 5778$\pm$3069 | 27 | 6791$\pm$4236 | 47 | 6771$\pm$3331 |
| 8 | 5333$\pm$3159 | 28 | 5675$\pm$3421 | 48 | 6058$\pm$3835 |
| 9 | 5948$\pm$3944 | 29 | 6619$\pm$3680 | 49 | 6721$\pm$3627 |
| 10 | 7729$\pm$3847 | 30 | 5740$\pm$2658 | 50 | 6289$\pm$4103 |
| 11 | 10302$\pm$4687 | 31 | 6335$\pm$3517 | 51 | 6324$\pm$3675 |
| 12 | 6103$\pm$3076 | 32 | 6388$\pm$3099 | 52 | 6230$\pm$3971 |
| 13 | 6720$\pm$3477 | 33 | 5629$\pm$3199 | 53 | 6222$\pm$3866 |
| 14 | 9509$\pm$4435 | 34 | 6912$\pm$3628 |  |  |
| 15 | 6095$\pm$3647 | 35 | 6043$\pm$3016 |  |  |
| 16 | 7259$\pm$3530 | 36 | 6071$\pm$3082 |  |  |
| 17 | 8051$\pm$4484 | 37 | 5556$\pm$2796 |  |  |
| 18 | 6224$\pm$3962 | 38 | 6669$\pm$4116 |  |  |
| 19 | 7438$\pm$3506 | 39 | 5850$\pm$3744 |  |  |
| 20 | 7567$\pm$4119 | 40 | 6059$\pm$3549 |  |  |

**Supplement Table 4. Average number of time point of each item across subjects in healthy controls.**

| Item No. | Mean$\pm$SD | Item No. | Mean$\pm$SD | Item No. | Mean$\pm$SD |
| --- | --- | --- | --- | --- | --- |
| 1 | 8322$\pm$4052 | 21 | 9899$\pm3954$ | 41 | 8792$\pm$5139 |
| 2 | 6223$\pm$2287 | 22 | 8409$\pm$3792 | 42 | 5821$\pm$3387 |
| 3 | 8399$\pm$3864 | 23 | 6795$\pm$3135 | 43 | 7619$\pm$4377 |
| 4 | 6809$\pm$3045 | 24 | 6734$\pm$2931 | 44 | 6897$\pm$3078 |
| 5 | 5505$\pm$2456 | 25 | 6064$\pm$5226 | 45 | 5639$\pm$3861 |
| 6 | 7756$\pm$3160 | 26 | 6459$\pm$3287 | 46 | 6416$\pm$2967 |
| 7 | 5899$\pm$3726 | 27 | 6573$\pm$3652 | 47 | 5615$\pm$2551 |
| 8 | 4706$\pm$2534 | 28 | 6505$\pm$3212 | 48 | 6626$\pm$2080 |
| 9 | 5554$\pm$3486 | 29 | 4944$\pm$3126 | 49 | 6455$\pm$2925 |
| 10 | 7756$\pm$2887 | 30 | 5780$\pm$3425 | 50 | 5906$\pm$3306 |
| 11 | 9480$\pm$4266 | 31 | 6523$\pm$2806 | 51 | 6335$\pm$3567 |
| 12 | 5434$\pm$2154 | 32 | 5964$\pm$2776 | 52 | 5504$\pm$3024 |
| 13 | 6336$\pm$3439 | 33 | 7201$\pm$5534 | 53 | 7093$\pm$4088 |
| 14 | 9472$\pm$4141 | 34 | 8036$\pm$8134 |  |  |
| 15 | 5988$\pm$3400 | 35 | 6313$\pm$3891 |  |  |
| 16 | 6694$\pm$3753 | 36 | 5795$\pm$3341 |  |  |
| 17 | 7817$\pm$3887 | 37 | 5053$\pm$3102 |  |  |
| 18 | 6098$\pm$3485 | 38 | 6502$\pm$3356 |  |  |
| 19 | 7476$\pm$3901 | 39 | 5757$\pm$4389 |  |  |
| 20 | 7934$\pm3258$ | 40 | 5378$\pm$3513 |  |  |
