# Supplementary material for: Questionnaires based on natural language processing elicit immersive ruminative thinking in ruminators: Evidence from behavioral responses and EEG data
Source: Front Neurosci. 2023 Mar 6;17:1118650. doi: 10.3389/fnins.2023.1118650 (PMC10025410; doi:10.3389/fnins.2023.1118650)
Supplement: Supplementary file 1 [file Data_Sheet_1.ZIP › Supplementary Figure-2023-1-25.docx]

**
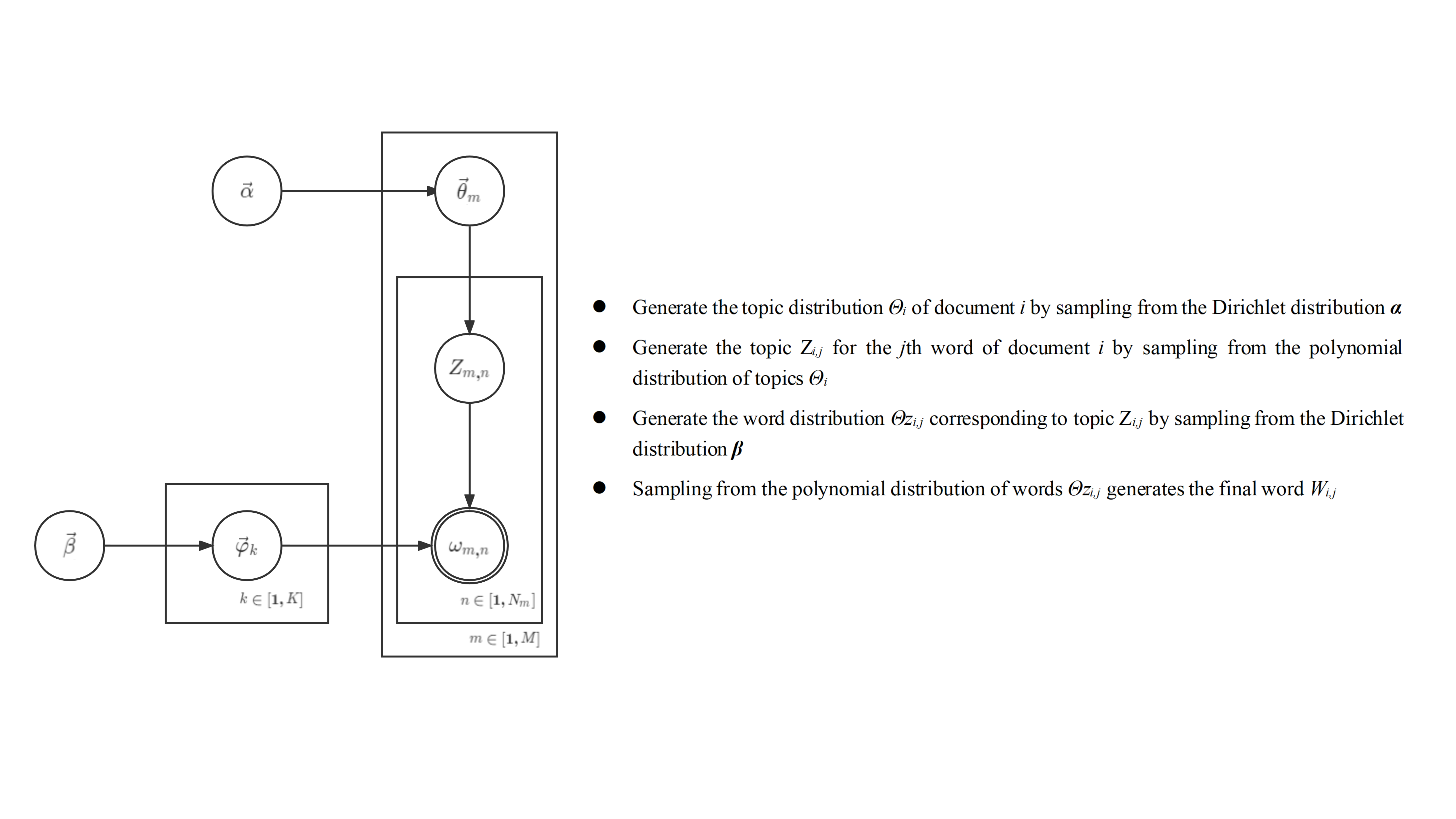
**

**Supplementary Figure 1. Graphical representation of the LDA model and document generation process.**

The analysis pipeline of the LDA model and the document generation process are shown in Supplementary Figure 1 (Blei, Ng, & Jordan, 2003). LDA is a three-level Bayesian probabilistic model and a document generation model with observable variables represented as double circles *w* and other latent variables represented as single circles. *α* is the Dirichlet parameter before each document topic distribution, *β* is the Dirichlet parameter before each topic word distribution, *Θ_ⅰ_* is the topic distribution of document *i* (sum of *Θ_ⅰ_* is 1.0), *φ_k_* is the word distribution of topic *k*, Z*_i,j_* is the topic of the *j*th word in document *i*, and *W_i,j_* is the specific word. Regarding the parameter settings of the LDA learning algorithm, in this study, *α* and *β* were set to 0.1 and 0.01 respectively, which are common settings in the literature (Hao, Zhang, Wang, & Gao, 2017).

Blei, D. M., Ng, A., & Jordan, M. I. (2003). Latent dirichlet allocation. *The Journal of Machine Learning Research.* doi: 10.1162/jmlr.2003.3.4-5.993

Hao, H., Zhang, K., Wang, W., & Gao, G. (2017). A tale of two countries: International comparison of online doctor reviews between China and the United States. *International Journal of Medical Informatics*, 99, 37-44. doi: 10.1016/j.ijmedinf.2016.12.007
